# Phosphorus spectroscopy in acute TBI demonstrates metabolic changes that relate to outcome in the presence of normal structural MRI

**DOI:** 10.1177/0271678X18799176

**Published:** 2018-09-18

**Authors:** Matthew G Stovell, Marius O Mada, T Adrian Carpenter, Jiun-Lin Yan, Mathew R Guilfoyle, Ibrahim Jalloh, Karen E Welsh, Adel Helmy, Duncan J Howe, Peter Grice, Andrew Mason, Susan Giorgi-Coll, Clare N Gallagher, Michael P Murphy, David K Menon, Peter J Hutchinson, Keri LH Carpenter

**Affiliations:** 1 Division of Neurosurgery, Department of Clinical Neurosciences, University of Cambridge, Cambridge, UK; 2 Wolfson Brain Imaging Centre, Department of Clinical Neurosciences, University of Cambridge, Cambridge, UK; 3 Department of Neurosurgery, Keelung Chang Gung Memorial Hospital, Chang Gung University College of Medicine, Taoyuan, Taiwan; 4 Department of Chemistry, University of Cambridge, Cambridge, UK; 5 Division of Neurosurgery, Department of Clinical Neurosciences, University of Calgary, Calgary, Alberta, Canada; 6 MRC Mitochondrial Biology Unit, University of Cambridge, Cambridge, UK; 7 Division of Anaesthesia, Department of Medicine, University of Cambridge, Cambridge, UK

**Keywords:** ^31^P magnetic resonance spectroscopy, adenosine triphosphate, clinical outcome, pH, traumatic brain injury (human)

## Abstract

Metabolic dysfunction is a key pathophysiological process in the acute phase of traumatic brain injury (TBI). Although changes in brain glucose metabolism and extracellular lactate/pyruvate ratio are well known, it was hitherto unknown whether these translate to downstream changes in ATP metabolism and intracellular pH. We have performed the first clinical voxel-based *in vivo* phosphorus magnetic resonance spectroscopy (^31^P MRS) in 13 acute-phase major TBI patients versus 10 healthy controls (HCs), at 3T, focusing on eight central 2.5 × 2.5 × 2.5 cm^3^ voxels per subject. PCr/γATP ratio (a measure of energy status) in TBI patients was significantly higher (median = 1.09) than that of HCs (median = 0.93) (p < 0.0001), due to changes in both PCr and ATP. There was no significant difference in PCr/γATP between TBI patients with favourable and unfavourable outcome. Cerebral intracellular pH of TBI patients was significantly higher (median = 7.04) than that of HCs (median = 7.00) (p = 0.04). Alkalosis was limited to patients with unfavourable outcome (median = 7.07) (p < 0.0001). These changes persisted after excluding voxels with > 5% radiologically visible injury. This is the first clinical demonstration of brain alkalosis and elevated PCr/γATP ratio acutely after major TBI. ^31^P MRS has potential for non-invasively assessing brain injury in the absence of structural injury, predicting outcome and monitoring therapy response.

## Introduction

Traumatic brain injury (TBI) is a major cause of death and disability worldwide, placing high demands on carers and resources.^1^ Neurosurgical and neurocritical care attempt to support the brain’s recovery through a critical period when the damaging pathophysiological processes of secondary brain injury occur. A key contributor to secondary brain injury is cerebral metabolic dysfunction. Acute TBI patients often show elevated concentration of extracellular lactate relative to pyruvate (high L/P ratio) despite showing seemingly adequate cerebral perfusion pressure and local brain tissue oxygen.^2,3^ This is attributed to a failure of mitochondrial function^4,5^ as L/P ratio represents reduced/oxidised nicotinamide adenine dinucleotide (NADH/NAD^+^) redox state.^6^

It is unclear whether this translates to a change in high-energy phosphates in the acute phase after severe TBI. ATP is principally produced by mitochondrial oxidative phosphorylation and is also synthesised in the cytosol via glycolysis. The brain also contains the high-energy phosphate species phosphocreatine (PCr), which is most abundant in tissues requiring energy in bursts, e.g. muscle and brain. PCr acts as a reserve that can be rapidly mobilised for fast recycling of ADP to ATP to drive cellular processes in times of high demand, thereby acting as a temporal buffer for ATP; and due to its greater free diffusion in the cytoplasm compared to ATP and ADP, it also serves as a spatial buffer, smoothing out spatial variations in cellular energy state.^7,8^ A high brain extracellular L/P ratio acutely after severe TBI implies diversion of pyruvate away from entry into the tricarboxylic acid (TCA) cycle to maintain flux through glycolysis, which is likely to limit oxidative phosphorylation. However, changes in brain energy storage, expressed as PCr/ATP ratio, have not previously been investigated within the human brain during the acute phase of injury.

**Figure 1.**
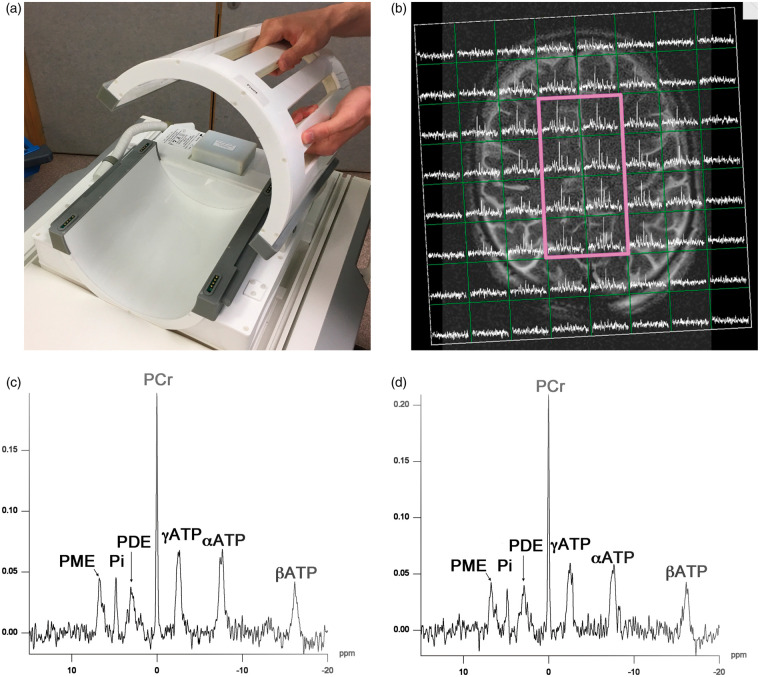
^31^P MRS acquisition. (a): Custom-built (PulseTeq Ltd) birdcage ^31^P head-coil for use on Siemens 3-Tesla magnetic resonance (MR) imaging scanner. The head-coil opens to facilitate use with patients. Image is courtesy of the Wolfson Brain Imaging Centre. (b): Example of axial T2 weighted MRI scan (Siemens 3 T) with ^31^P MR spectra overlaid in a grid of voxels using the custom head-coil (Pulseteq Ltd) in a healthy control. The eight central voxels within the bold magenta outline were used for data analysis. (c and d): Example ^31^P spectrum from a central voxel in a healthy control (c) and traumatic brain injury patient (d), with 200 ms Hanning filter. Metabolite peaks labelled. PE: phosphomonoesters; Pi: inorganic phosphate; PDE: phosphodiesters; PCr: phosphocreatine; ATP: adenosine triphosphate.

Here, we demonstrate the first voxel-based in vivo phosphorus magnetic resonance spectroscopy (^31^P MRS) study of brain energetics in severe acute TBI in humans. Steady-state in vivo ^31^P MRS is a non-invasive technique for interrogating high-energy phosphate species, including ATP and PCr, inorganic phosphate (Pi) and brain pH, by using a dedicated ^31^P head coil (Figure 1) – using relative concentrations of these metabolites (rather than rates of flux) to report the state of brain energy metabolism. This is the first voxel-based in vivo chemical shift imaging ^31^P MRS study of brain energetics and pH in humans in the acute phase of moderate-severe TBI.^1^ Our state-of-the-art clinical MR scanning has yielded high-quality well-resolved ^31^P spectra with a good baseline, enabling reliable measurement of metabolites in multiple voxels per patient. 3D localisation has allowed accurate assessment of grey/white matter ratio and radiological injury of each voxel. Our scanner’s location directly adjacent to the neurosciences critical care unit has enabled us to focus on severe TBI patients in the acute phase, whilst fully sedated and ventilated.

We report changes in brain energy metabolism and intracellular pH in the acute phase of major TBI, evaluate how these data relate statistically to patients’ clinical outcomes after six months and identify altered brain metabolism in brain that appears uninjured on MRI sequences sensitive to pathology.

## Materials and methods

### Study design, patients and healthy controls

We recruited patients (aged over 16 years) who had sustained moderate or severe TBI (defined as cranial trauma with consistent CT scan findings and a post-resuscitation Glasgow Coma Scale (GCS) ≤12) that required sedation and mechanical ventilation for intracranial hypertension and airway protection (Table 1) – which we describe as ‘major’ TBI. Two patients who presented initially with moderate TBI (GCS 10) deteriorated during resuscitation to GCS ≤ 8. Patients were treated using our standard TBI management protocols including: endotracheal intubation, ventilation, sedation, muscular paralysis and maintenance of blood sugar (serum glucose) concentration within the target range 4–10 mmol/L.^9^ Scans were in the acute phase as soon as patients’ intracranial pressure permitted them to be laid supine, whilst still requiring full sedation, ventilation and monitoring for control of intracranial hypertension. Of our 13 patients, 9 were scanned within a week of primary injury and 4 within two weeks of injury because their persistent brittle intracranial hypertension precluded them from being laid supine for MR any sooner. Arterial blood samples were taken and analysed for pH and partial pressure of CO_2_ (PaCO_2_) before and after patients’ scans, and the average of the two results was calculated. Informed written assent was obtained from the patients’ relatives, and age-group-matched healthy controls were recruited locally with their informed written consent. The study conformed to the spirit and the letter of the Declaration of Helsinki. Protocol approval was by the National Research Ethics Service (NRES) Committee East of England – Cambridge Central (REC Reference No. 11/EE/0463). Patients’ six-month follow-up included Glasgow Outcome Scale Extended^10^ (GOS-E) scoring, assessed without knowledge of study results. Outcome was dichotomised into upper severe disability or better (GOS-E ≥ 4; independent at home) and lower severe disability or worse (GOS-E ≤ 3; dependent at home, vegetative or dead), as in other recent studies.^11,12^

**Table 1.** Demography of traumatic brain injury patients.

Subject number	Age group	Sex	Injury mechanism	Brain injury	GCS total	GCS E, V, M	Days from TBI	GOS-E six months	Outcome
P01	20–34	M	Assault	EDH, brain contusions	8	E1V2M5	5	3	Unfav.
P02	50–65	F	Fall from height	ASDH, ICH	7	E2V1M4	3	5	Fav.
P03	35–49	M	Assault	DAI, hypoxia	8	E3V4M1	10	2	Unfav.
P04	50–65	M	RTC	EDH, ICH	3	E1V1M1	4	1	Unfav.
P05	50–65	M	Presumed assault	ASDH, brain contusions	10	E4V2M6	7	2	Unfav.
P06	20–34	M	RTC	brain contusions	6	E1V1M4	4	5	Fav.
P07	35–49	M	Fall from height	ASDH, brain contusions	8	E2V1M5	5	5	Fav.
P08	35–49	M	RTC	ASDH	6	E1V1M4	11	5	Fav.
P09	35–49	M	Assault	ASDH, EDH, brain contusions	6	E1V2M3	4	1	Unfav.
P10	50–65	F	RTC	EDH, brain contusions	10	E3V1M6	6	1	Unfav.
P11	20–34	F	RTC	brain contusions	5	E1V2M2	14	4	Fav.
P12	20–34	M	RTC	brain contusions	7	E1V1M5	13	5	Fav.
P13	50–65	M	Assault	ASDH, brain contusions	4	E1V1M2	4	5	Fav.

Note: Age group in years. GCS denotes highest GCS at presentation to emergency services. Patients P5 and P10 presented as only moderately drowsy, but then rapidly deteriorated, requiring sedation, intubation, ventilation and surgery for their TBI followed by a period of intracranial multimodality monitoring and treatment for intracranial hypertension. Patients P03, P08, P11 and P12 had persistently high ICP that was difficult to control and were scanned as soon as they could tolerate lying flat. At the time of the scans, they still required sedation, ventilation and active ICP control, thus still representing the ‘acute phase’ after TBI.

M: male, F: female, RTC: road traffic collision, GCS: Glasgow Coma Scale score, E: eye response, V: verbal response, M: motor response, L: left, R: right, EDH: extradural haematoma, ASDH: acute subdural haematoma, ICH: intracerebral haematoma, GOS-E: Glasgow outcome score (Extended), Outcome: favourable (Fav.) GOS-E ≥ 4; unfavourable (Unfav.) GOS-E ≤ 3.

### Magnetic resonance spectroscopy

We used 3 Tesla Siemens (Trio and Verio) scanners with a custom ^31^P head-coil (PulseTeq Ltd, Chobham, UK) (Figure 1(a)) with a birdcage design that opens to facilitate use with our patients, measuring ATP, PCr, Pi and other phosphorus-containing species. Spectra were acquired using oblique two-dimensional single slice chemical shift imaging (2D CSI) with 2.5 × 2.5 × 2.5 cm^3^ voxels, flip angle 90 degrees, TR 4000 ms, TE 2.3 ms and 3000 Hz bandwidth. Repeats were performed for 30 (19 min) or 60 (35 min) averages. Weighted sampling of k-space was performed. Patients also received ^1^H clinical MR imaging, including sequences sensitive for detecting injury: fluid-attenuated inversion recovery (FLAIR) and susceptibility weighted imaging (SWI). ^31^P spectra were filtered with a 200 ms Hanning filter, fitted and peak areas computed using Siemens Syngo software. Absolute concentrations are difficult to accurately quantify with in vivo clinical ^31^P MRS, so we used the ratio of PCr to γATP signal intensities of the fitted spectra as our primary measure of high-energy phosphate metabolic status.^13,14^ We employed γATP, the ‘cleanest’ of the three ATP signals. We did not use the βATP peak as it is not reliably excited due to being at the edge of our excitation bandwidth at 3T, and we did not use the αATP peak as it is incompletely resolved from NAD and NADH. We did not attempt to adjust ATP values for the presence of ADP as it is naturally much less abundant, e.g. ATP ≈ 3 mmol/L, ADP < 100 µmol/L^15^ and mostly MR-invisible.^16,17^ To indicate whether variation in PCr/γATP ratio was driven principally by changes in PCr or γATP, we assessed the ratio of PCr to total-mobile-phosphate (defined as the combined signals PCr plus γATP plus Pi) and the ratio of γATP to total-mobile-phosphate.^13,14^ The PCr, γATP and Pi resonances together represent the total mobile high-energy phosphate pool involved directly with ATP metabolism. Intracellular pH was calculated from the chemical shift difference between PCr and Pi, using an established equation.^18,19^

Voxel tissue type (grey matter, white matter and CSF) was segmented using FAST (FMRIB’s Automated Segmentation Tool) processing of 3D T1W gradient echo sequences (MP RAGE) in FSL (FMRIB software, Oxford, UK). Regions of FLAIR and SWI radiological injury were reviewed and manually mapped. Ratios of grey matter/white matter and radiologically visible injury were then calculated for each voxel using Matlab software (MathWorks, Natick, MA). Our primary inclusion criterion for data analysis was that voxels contained at least 90% brain tissue; voxels with less than 90% brain tissue were excluded at the outset. A sub-analysis of ‘radiologically-normal’ brain was performed by further excluding voxels with more than 5% injury on FLAIR or SWI. As part of a separate biochemical study, nine voxels were supplemented (via a microdialysis catheter, prior to scan) with succinate or glucose, and therefore were excluded from the present data analysis.^20^

### Statistical analysis

Statistical analysis was performed in R (www.r-project.org). Comparison of PCr/γATP, PCr/total-mobile-phosphate, γATP/total-mobile-phosphate and pH between healthy controls and TBI patients was performed with subject-mean data, using Mann-Whitney U test, and repeated using a linear mixed effects model (‘lme’ in R package nlme^21^) of pooled data, which accounts for ‘clustering’ of data as each subject contributes multiple voxels. Further comparisons of high-energy phosphate ratios and pH between TBI patients with ‘favourable outcome’ (GOS-E ≥ 4) and ‘unfavourable outcome’ (GOS-E ≤ 3) were also performed with Mann-Whitney U of subject-mean data and lme of individual voxels, using generalised linear hypothesis tests (glht) with Bonferroni correction when healthy controls were included in the model of patient outcome. Voxel grey matter/white matter ratio was included as a covariate in all mixed effect models. The relationship between brain pH and PCr/ATP ratio, arterial blood pH and PaCO_2_ was assessed with Spearman’s rank correlation. Potential confounders/nuisance variables were explored using Spearman’s rank correlation and Mann-Whitney U tests. Results quoted for outcome are group medians of within-patient means. Graphs were plotted with R and Origin.

### Data availability

This study’s datasets are not publicly available because of patient confidentiality, but anonymised data are available from Prof Peter Hutchinson (the Principal Investigator), on reasonable request.

## Results

In vivo ^31^P MRS data were available for a total of 90 voxels from 13 sedated, ventilated TBI patients (nine male, four female), median age 42 years, range 24–65 years, and 80 voxels from 10 age-group and sex-matched healthy control subjects. Patients’ demography is given in Table 1, and healthy controls are given in Supplementary Table 1. No clinical complications resulted from MRS. An example scan is shown in Figure 1(b) and ^31^P spectra examples are shown in Figure 1(c) and (d). Summary ^31^P MRS data are given in Table 2 and individual patient data are given in Table 3. Our scans were performed as early after injury as clinically feasible, whilst patients still required deep sedation and neurocritical care, so were regarded as within the ‘acute phase’ of injury.

**Table 2.** Summary of ^31^P MRS results: group medians and interquartile ranges of within-subject means.

	Number subjects	Injured voxels	PCr/γATP Median (IQR)	PCr/total Median (IQR)	γATP/total Median (IQR)	pH Median (IQR)
TBI: all patients	13	Including	1.09 (1.04–1.20)	0.47 (0.46–0.48)	0.44 (0.40–0.45)	7.04 (7.02–7.05)
		Excluding	1.07 (1.06–1.21)	0.47 (0.46–0.49)	0.44 (0.40–0.45)	7.03 (7.01–7.05)
Healthy controls	10	NA	0.93 (0.86–0.96)	0.42 (0.41–0.43)	0.46 (0.45–0.48)	7.00 (6.99–7.00)
p (TBI vs. HC)		Including	<0.0001	<0.0001	0.0009	0.042
		Excluding	<0.0001	<0.0001	0.001	0.055
TBI: favourable outcome	7	Including	1.07 (1.04–1.10)	0.48 (0.46–0.48)	0.44 (0.44–0.45)	7.02 (7.00–7.03)
		Excluding	1.07 (1.06–1.09)	0.47 (0.46–0.48)	0.44 (0.44–0.45)	7.02 (7.00–7.03)
TBI: unfavourable outcome	6	Including	1.14 (1.07–1.22)	0.47 (0.46–0.49)	0.41 (0.39–0.42)	7.07 (7.04–7.14)
		Excluding	1.14 (1.06–1.24)	0.48 (0.47–0.49)	0.41 (0.40–0.44)	7.07 (7.03–7.16)
p (Fav vs. Unfav.)		Including	0.3	0.9	0.04	0.03
		Excluding	0.3	0.9	0.06 [0.04]	0.057 [0.008]

Note: ^31^P magnetic resonance spectroscopy (MRS) measurements of pH, phosphocreatine (PCr), adenosine triphosphate (***γ***ATP) and ‘total’ mobile phosphate (PCr + ***γ***ATP + inorganic phosphorus) ratios in 13 patients suffering from acute major traumatic brain injury (TBI) and 10 age-group matched healthy controls (HC). Results represent group medians of within-subject means, with interquartile ranges (IQR) in parentheses (curved brackets); for each individual subject’s mean values see Table 3. Voxels were considered injured if they contained ≥5% radiological injury on fluid-attenuated inversion recovery (FLAIR) or susceptibility weighted injury (SWI) MR sequences. Favourable outcome (Fav) defined as six-month Glasgow outcome score (extended) (GOS-E) ≥4, and unfavourable outcome (Unfav.) GOS-E ≤ 3. Statistical analysis performed using a linear mixed model in R (lme in package nlme), adjusting for voxel grey matter/white ratio. Inclusion of healthy controls and analysis of the linear model using glht (results in Figure 4) found the difference in ***γ***ATP/total-mobile-phosphate to be statistically significant (results in square [ ] brackets), but did not affect the significance of other measurements.

**Table 3.** ^31^P MRS data acquired from TBI patients and healthy controls.

Subject ID	PCr/γATP mean	PCr/γATP S.D.	PCr/total mean	PCr/total S.D.	γATP/total mean	γATP/total S.D.	pH mean	pH S.D.	No. of voxels
P01	1.06	0.11	0.47	0.03	0.45	0.02	7.03	0.09	7
P02	1.10	0.17	0.48	0.04	0.44	0.03	7.02	0.03	7
P03	1.41	0.13	0.52	0.04	0.37	0.03	7.29	0.07	7
P04	1.00	0.12	0.42	0.06	0.42	0.04	7.15	0.09	7
P05	1.09	0.23	0.46	0.06	0.42	0.04	7.10	0.07	4
P06	1.07	0.14	0.48	0.04	0.45	0.02	7.01	0.07	6
P07	1.03	0.13	0.45	0.03	0.45	0.02	6.98	0.02	8
P08	1.21	0.13	0.48	0.04	0.40	0.04	7.02	0.06	8
P09	1.20	0.04	0.47	0.02	0.39	0.02	7.05	0.05	6
P10	1.23	0.09	0.49	0.03	0.40	0.03	7.04	0.02	7
P11	1.04	0.05	0.46	0.01	0.44	0.02	7.04	0.02	7
P12	1.04	0.08	0.46	0.03	0.44	0.01	7.04	0.07	8
P13	1.10	0.28	0.50	0.06	0.46	0.05	6.96	0.06	8
H01	0.95	0.07	0.42	0.02	0.45	0.01	6.99	0.02	8
H02	0.74	0.07	0.37	0.02	0.50	0.02	7.00	0.02	8
H03	0.92	0.04	0.42	0.03	0.46	0.03	7.00	0.04	8
H04	0.98	0.05	0.44	0.02	0.45	0.01	7.00	0.01	8
H05	0.82	0.05	0.40	0.02	0.49	0.03	6.99	0.02	8
H06	0.99	0.04	0.45	0.01	0.45	0.01	6.97	0.03	8
H07	0.95	0.06	0.43	0.02	0.45	0.01	7.00	0.01	8
H08	0.88	0.04	0.42	0.02	0.48	0.01	6.99	0.04	8
H09	0.86	0.04	0.41	0.01	0.48	0.01	7.00	0.03	8
H10	0.97	0.04	0.43	0.03	0.45	0.03	7.03	0.03	8

Note: Individual subject ^31^P magnetic resonance spectroscopy (MRS) measurements of pH, phosphocreatine (PCr), adenosine triphosphate (***γ***ATP) and ‘total’ mobile phosphate (PCr + ***γ***ATP + inorganic phosphorus (Pi)) ratios derived from the central eight voxels of 13 TBI patients (P1–P13) and 10 age-group-matched healthy controls (H1–H10). As part of a separate biochemical study, some patients had microdialysis catheters supplemented with either succinate or glucose, so a voxel was excluded from analysis (P01, P02, P03, P04, P05, P06, P10 and P11). Two subjects’ CSI grids were positioned to capture superficially placed microdialysis catheters, excluding a further one (P06 and P09) and three (P05) voxels that represented <90% brain tissue.

### ^31^P MRS: metabolic changes in acute TBI

**Figure 2.**
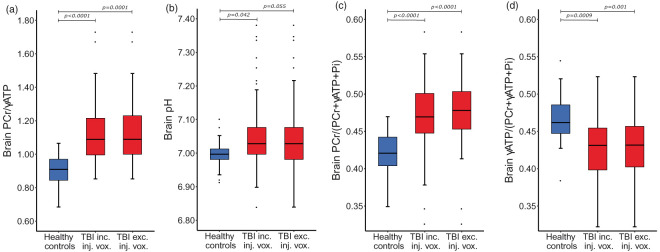
PCr/γATP, pH, PCr/total-mobile-phosphate and γATP/total-mobile-phosphate changes following TBI. Box-and-whisker plots of ^31^P magnetic resonance spectroscopy measurements in the central eight voxels of healthy controls (in blue, 80 voxels) TBI patients (in red, 90 voxels: ‘TBI inc. inj. vox.’), and TBI patients after excluding voxels that contained >5% radiological injury on FLAIR or SWI sequences (in red, 73 voxels: ‘TBI exc. inj. vox.). There was a statistically significant difference in PCr/γATP ratio (a), pH (b), PCr/total-mobile-phosphate (c) and ***γ***ATP/total-mobile-phosphate (d) between TBI patients and age-group-matched healthy controls. The statistically significant differences persisted when injured voxels were excluded from analysis, except for brain pH (b, p* = *0.055). Statistical analysis was performed using a linear mixed effects model (lme in R package nlme) that included voxel grey matter/white matter ratio as a covariate, p-values quoted in the figure. FLAIR: fluid-attenuated inversion recovery; SWI: susceptibility weighted imaging; PCr: phosphocreatine; γATP: adenosine triphosphate; total-mobile-phosphate: PCr + γATP + inorganic phosphate.

**Figure 3.**
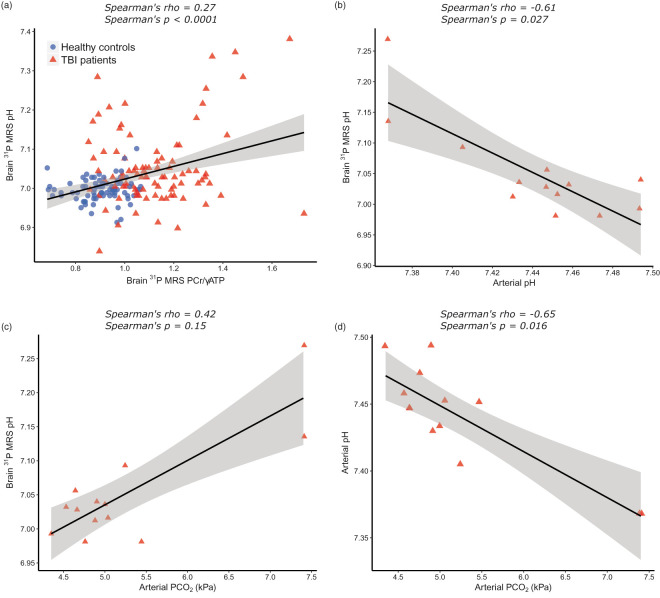
Scatter plots showing associations between brain pH, PCr/γATP, arterial pH and arterial PaCO_2_ following TBI. (a): Brain pH and PCr/γATP ratio of combined voxels (n = 170) from healthy controls (blue circles) and TBI patients (red triangles) demonstrate a positive correlation between the two (rho = 0.27, p < 0.0001). (b): Individual patient mean brain pH, measured using ^31^P MRS, is inversely correlated with patient arterial blood pH, measured with bedside blood gas analyser (rho = −0.61, p = 0.027). (c): There was an inverse correlation between arterial pH and PaCO_2_, as expected (rho = −0.65, p = 0.016). (d): there was no statistically significant relationship between arterial blood PaCO_2_ and brain pH. All correlations are Spearman’s ranked correlation coefficient with solid linear regression line; 95% confidence interval denoted by grey-shaded area.

We first compared the high-energy phosphate ratios and pH of TBI patients and age-group matched healthy controls with Mann-Whitney U test, using each subject’s mean value calculated from the central eight voxels of their brains. PCr/γATP was higher in TBI patients (Figure 2(a)): median (and interquartile range, IQR) PCr/γATP was 1.09 (1.04–1.20) in TBI patients and 0.93 (0.86–0.96) in healthy controls (p < 0.0001). PCr/total-mobile-phosphate was also higher in TBI patients (Figure 2(c)): median PCr/total-mobile-phosphate was 0.47 (0.46–0.48) in TBI patients and 0.42 (0.41–0.43) in healthy controls (p < 0.0001); whereas γATP/total-mobile-phosphate was lower in TBI patients (Figure 2(d)): median γATP/total-mobile-phosphate was 0.44 (0.40–0.45) in TBI patients and 0.46 (0.45–0.48) in healthy controls (p = 0.0003). TBI patients’ brain pH was more alkaline than that of healthy controls (Figure 2(b)): median 7.04 (7.02–7.05) in TBI patients; and 7.00 (6.99–7.00) in healthy controls (p = 0.006). Intracellular pH correlated significantly with PCr/γATP (Figure 3(a)). This association was not strong (Spearman’s correlation coefficient rho* = *0.27), although highly statistically significant (p < 0.0001). There was a statistically significant inverse correlation between brain pH measured by ^31^P MRS and arterial blood pH measured by blood gas analyser (Spearman’s rho = −0.61, p = 0.027) (Figure 3(b)). Arterial pH ranged from 7.37 to 7.49: nine patients’ results were within accepted normal physiological range (7.35–7.45), four patients were slightly alkalotic. There was no statistically significant correlation between brain pH and arterial blood PaCO_2_ (Spearman’s rho = 0.42, p = 0.15) (Figure 3(c)).

We repeated the analysis with a linear mixed effects model using all (n = 170) voxels. This again found a statistically significant difference in PCr/γATP, PCr/total-mobile-phosphate, γATP/total-mobile-phosphate and pH between TBI patients and healthy controls, verifying Mann-Whitney U results (Table 2, Figure 2).

There was greater variation in pH and high-energy phosphate ratios between TBI patients than between healthy controls (Table 2). The variability within each subject’s eight voxels was also greater in TBI patients (Table 3). Exploration of potential confounding variables found no correlation between pH or PCr/γATP ratio and either patient age, or interval between injury and MRS scanning, nor was a difference found when patients were dichotomised into those less than or more than seven days after injury (Mann-Whitney U). Grey matter/white matter ratio was not significantly different between TBI patients and healthy controls (p = 0.26, Mann-Whitney), nor did it reach significance as a covariate in linear mixed model analysis of PCr/γATP, PCr/total-mobile-phosphate or pH. However, it was a statistically significant covariate in linear mixed model analysis of γATP/total-mobile-phosphate (p = 0.03), and as it can affect ratios of high-energy phosphates in the healthy brain,^14,22^ it was included in all mixed effects model analysis. Inclusion of scanner type (Siemens Trio or Verio) in the mixed effects models did not affect significance.

### ^31^P MRS: metabolic changes by outcome

**Figure 4.**
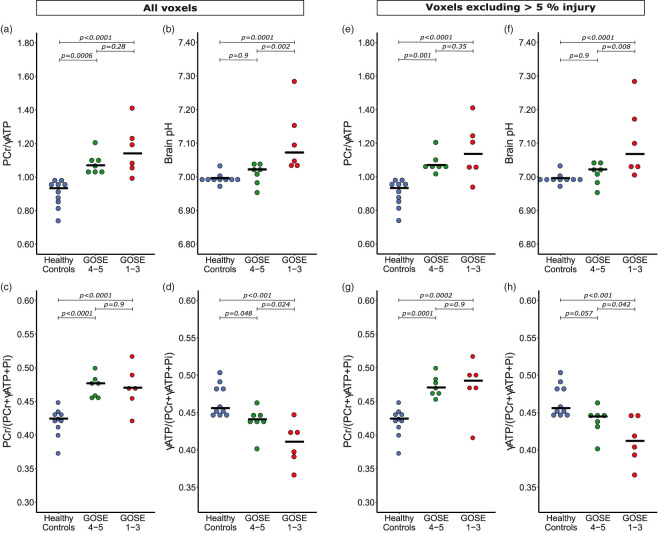
PCr/γATP, pH, PCr/total-mobile-phosphate and γATP/total-mobile-phosphate changes following TBI by patient outcome – including and excluding injured voxels. Dot-plots of ^31^P magnetic resonance spectroscopy measurements, with each point representing subject mean result, split by patient outcome at six months: healthy controls; favourable outcome (GOS-E ≥ 4); and unfavourable outcome (GOS-E 1–3). (a–d) include all patient voxels, (e–h) only include voxels that contained <5% radiological injury on fluid-attenuated inversion recovery (FLAIR) or susceptibility weighted imaging (SWI) sequences. (a): PCr/γATP ratio was raised in both patient groups compared to healthy controls. (b): brain pH was significantly higher in patients with an unfavourable outcome than healthy controls and patients with a favourable outcome, who did not observe a change in their brain pH. (c): PCr/total-mobile-phosphate ratio was elevated in both patient outcome groups equally. (d): γATP/total-mobile-phosphate ratio was significantly lower in TBI patients potentially scaled to outcome, with a lower γATP/total-mobile-phosphate ratio being found in patients with a worse outcome. After excluding voxels with ≥5% injury on FLAIR and SWI sequences, the statistical significance remained with differences in PCr/γATP ratio (e)*,* brain pH (f) and PCr/total-mobile-phosphate ratio (g). The statistical significant difference in γATP/total-mobile-phosphate ratio between healthy controls and patients with a favourable outcome was narrowly lost (p = 0.057) (h). Statistical analysis was performed using a linear mixed model in R (package nlme) that included voxel grey matter/white matter ratio as a covariate, using generalised linear hypothesis tests (glht) with Bonferroni correction of the mixed effects model for inter-group comparisons. Statistical significance is indicated by bars in figures. PCr: phosphocreatine; γATP: adenosine triphosphate; total-mobile-phosphate: PCr + γATP + inorganic phosphate.

**Figure 5.**
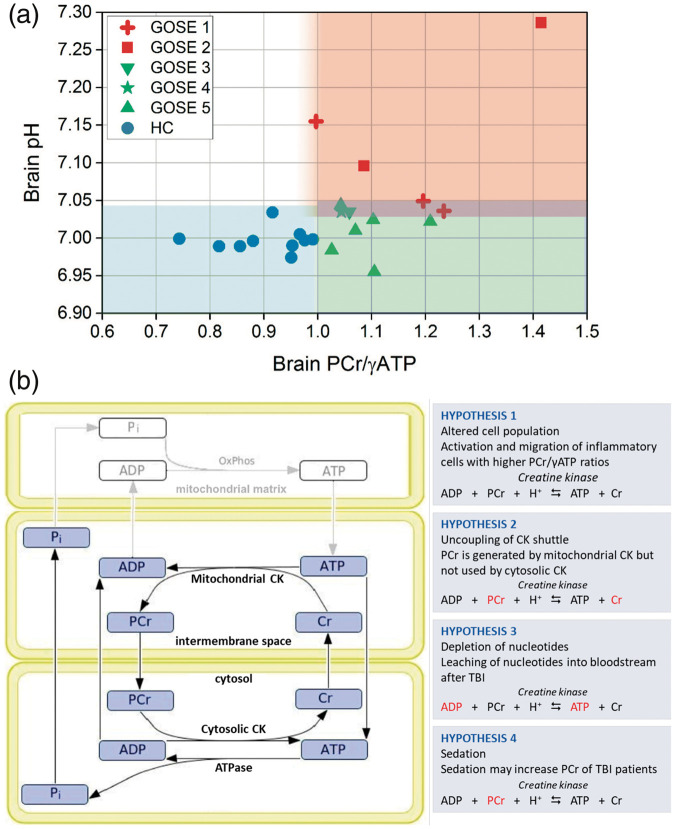
Hypotheses as possible explanations of our ^31^P MRS results, and scatter plot showing relationship between brain PCr/ATP, pH and patient outcome*.* (a): Scatter plot of intracellular pH vs. PCr/γATP, with data points differentiated for healthy controls and patients’ GOS-E outcome scores. Each data point represents the mean values for one subject. (b): hypotheses for changes observed in our sedated TBI patients, that may coincide with each other. The schematic of the compartmentalised creatine kinase system is adapted from Hettling and van Beek (2011, PLoS Comput Biol 7(8): e1002130, published Open Access © the Authors). ADP: adenosine diphosphate; ATP: adenosine triphosphate; Cr: creatine; CK: creatine kinase; PaCO_2_: arterial partial pressure of CO_2_; PCr: phosphocreatine; Pi: inorganic phosphate.

To establish whether the above changes were a marker of cellular stress and injury, or an appropriate compensatory response representing repair and recovery, we compared PCr/ATP and pH of patients with favourable outcome to those with unfavourable outcome six months after scanning using both Mann-Whitney U of subject means and linear mixed model of individual voxels. Patient outcome ranged between GOS-E-1 and GOS-E-5 and was dichotomised into favourable and unfavourable outcomes (see Materials and methods section). There was no statistically significant difference in PCr/γATP between patients with favourable and unfavourable outcomes (p > 0.2) or PCr/total-mobile-phosphate (p = 0.9) by either method. There was a trend for patients with an unfavourable outcome to have a lower γATP/total-mobile-phosphate than patients with a favourable outcome, narrowly missing significance by Mann-Whitney U (p = 0.051), but significant by mixed model analysis (p = 0.037). Brain alkalosis (pH) was higher in patients with unfavourable outcome than patients with favourable outcome using both analytical methods (p < 0.03) (Table 2). Furthermore, to ascertain if the biochemical changes observed in TBI patients (relative to healthy controls) were present in both TBI outcome groups, we then included healthy controls in our model (Figure 4). Compared to healthy controls, PCr/γATP and PCr/total-mobile-phosphate was elevated in patients with favourable outcome (p < 0.001) and patients with unfavourable outcome (p < 0.0001), with no difference between the two patient groups. γATP/total-mobile-phosphate was lower in patients both with favourable outcome (p = 0.048) and unfavourable outcomes (p < 0.0001) compared to healthy controls, with a greater fall found in patients with unfavourable outcome (p = 0.024) using this method of analysis. Brain pH was found to only be alkalotic in patients with unfavourable outcome (p = 0.0001), and was not significantly different between patients with favourable outcome and healthy controls (p = 0.9). Adding healthy controls to the mixed effects model did not diminish the earlier differences that were significant between patients with favourable and unfavourable outcomes. A scatter plot of intracellular pH vs. PCr/γATP with data points differentiated for healthy controls and individual GOS-E patient outcomes (Figure 5(a)) clearly shows that whilst PCr/γATP elevation was a generality in patients, alkalosis was only in those with worst outcomes.

### ^31^P MRS: metabolic changes in the absence of visible tissue injury

To ascertain whether brain alkalosis and elevated PCr/γATP occurred generally in the traumatised brain, rather than being limited to tissue visibly injured on MRI, we repeated the data analysis excluding voxels with more than 5% injury on FLAIR or SWI. This revealed the same pattern, although some statistical significance weakened (see Figure 2 and Table 2): There was still a difference in within-subject mean PCr/γATP, PCr/total-mobile-phosphate and γATP/total-mobile-phosphate between all TBI patients and healthy controls by Mann-Whitney U and mixed effects model analysis (p < 0.02). The difference in pH between patients and healthy controls remained significant by Mann-Whitney (p = 0.010), but narrowly missed significance by mixed effects model (p = 0.055). Comparing patients with favourable and unfavourable outcomes, there was still no significant difference in PCr/γATP or PCr/total-mobile-phosphate. The difference in γATP/total-mobile-phosphate narrowly lost significance, both by lme (p = 0.06) and by Mann-Whitney (p = 0.18). The difference in pH between patients with favourable outcome and unfavourable outcome narrowly lost significance by Mann-Whitney (p = 0.10) and lme comparing only the two patient outcome groups (p = 0.057), but remained significant when healthy control subjects were included in the mixed effects model (Figure 4).

## Discussion

We reveal significant changes in brain high-energy phosphate metabolism (elevation of PCr/ATP) and brain pH (alkalosis) in acute-phase major TBI patients, which appear to be related to clinical outcome. These differences persist when we excluded voxels containing structural abnormalities on MRI sequences sensitive for detecting injury following TBI – suggesting fundamental changes in energy state and acid–base balance occur in both visibly injured, and radiologically ‘uninjured’ acutely traumatised brain. Increases in patients’ PCr/ATP appear driven by changes in both PCr (rise in PCr/total-mobile-phosphate) and ATP (fall in ATP/total-mobile-phosphate) – but it is unclear whether these represent a single joint phenomenon, or two independent processes. Our findings contrast with the expectation of a low PCr/ATP ratio seen in animal studies of acute TBI,^23^ and an expectation of brain ‘acidosis’ reported extracellularly in acute TBI patients^24,25^ and intracellularly in hyper-acute animal TBI models.^23,26^ However, they are consistent with changes found in the subacute/chronic phase of TBI in humans,^27^ and sub-acutely after severe injury in rodent TBI models.^23,26^ Therefore, a high brain PCr/ATP and pH are not only recovery phase abnormalities of biochemistry, but are present in the acute phase of secondary injury after trauma. We hypothesise several explanations (Figure 5(b)), discussed below.

### Change in PCr/ATP energy state following TBI

Quantification of absolute metabolite concentrations using in vivo ^31^P MRS is challenging, and thus the PCr/ATP ratio is typically used to characterise cellular energy state. A high PCr/ATP ratio is interpreted as a tissue possessing greater energy reserve due to the relative abundance of the energy-replenishing PCr species relative to the active ATP species.^28^ Conventionally, when ATP synthesis is running normally, the cell’s PCr store is well stocked; when ATP synthesis is struggling to meet demand, the PCr store runs down.

The higher PCr/ATP in acute phase TBI, at face value, implies a ‘better-stocked’ store of energy in patients than controls, which might seem counter-intuitive given findings of other studies of adverse brain metabolism following major TBI: including metabolic rate of glucose,^29^ extracellular L/P ratio^2^ and ^31^P MRS experimental TBI models in animals.^30^ Indeed, findings of animal studies hyper-acutely after experimental injury showed decreased PCr with unchanging ATP (therefore lower PCr/ATP ratio),^23,26,30^ whereas we found high PCr/total-mobile-phosphate and low ATP/total-mobile-phosphate suggesting that both an increase in PCr and a fall in ATP occurred in our TBI patients’ brains.

The high PCr/ATP and PCr/total-mobile-phosphate in our TBI patients may be due to a change in the brain’s cell population (Hypothesis 1 in Figure 5(b)). A neuroinflammatory cascade occurs after major TBI, with proliferation and migration of glia and inflammatory cells, e.g. macrophages.^31^ Maturation and activation of macrophages increase their PCr concentration, producing a PCr ‘reservoir’ not seen in non-activated monocytes in the circulation.^32^ Glia possess a naturally higher PCr/ATP ratio than neurons^33,34^ and reserve capacity for ATP generation by increasing glycolysis of glycogen stores, or through autophagy.^34–38^ A higher PCr/ATP ratio (relative to healthy controls) was reported in white matter in the subacute period following TBI, attributed to glial proliferation (‘reactive gliosis’).^27^ We propose that such upregulation of resident glial metabolism and activation and migration of inflammatory cells with higher PCr/ATP ratios, either from the bloodstream or through activation of resident microglia, are the most important contributors to the elevated PCr/ATP ratio in our study.

The rise in PCr/ATP and fall in ATP/total-mobile-phosphate may also represent a degree of neural tissue energy failure occurring independently of brain cell population changes and/or glial activation (Hypothesis 2, Figure 5(b)). Mammalian cells work hard to maintain ATP homeostasis, even when stressed,^39–41^ so the apparent fall in ATP implied by a lower ATP/total-mobile-phosphate indicates more extreme metabolic dysfunction, and is most evident in patients with unfavourable outcomes. The high PCr/total-mobile-phosphate we found is surprising, as PCr would be expected to regenerate ATP. However, elevated PCr/ATP may represent a combined result from two different cell populations in the traumatised brain: cells that are more sensitive to injury, such as neurons, experiencing energy failure with low ATP; and activated glia more resistant to injury, and inflammatory cells which may have a relatively higher concentration of PCr. Such concept of differentially viable cellular moieties was proposed in a subacute study of large-territory stroke,^42^ which may share similar pathophysiology with patients who have sustained a major traumatic injury. Additionally, leaching of adenine bases from the injured brain^43,44^ may contribute to the finding of low ATP together with elevated PCr; as if high-energy phosphates were generated in the absence of sufficient adenine bases, the CK equilibrium may be shifted to a higher PCr/ATP ratio. However, we regard this explanation less likely, as we would not expect selective ATP loss without loss of PCr and Cr from the traumatised brain.

Uncoupling of the mitochondrial-cytosolic CK system (Hypothesis 3 in Figure 5(b)) may also contribute to high PCr/ATP in TBI patients. PCr is synthesised in the mitochondrial inter-membrane space by octameric mitochondrial CK (Mi-CK), and used in the cytosol by the dimeric, cytosolic form of CK (BB-CK). If TBI causes greater disruption or injury to cerebral BB-CK than to Mi-CK, or disruption of PCr and Cr shuttling between the two in the cytosol and mitochondria, PCr may be produced but not used, resulting in elevated PCr/ATP from accumulation of PCr and consumption of ATP. Although PCr (211 Da) is regarded as readily diffusible in cells due to its size and charge,^7,8^ the restricted diffusion of a similar sized molecule, N-acetylaspartate (NAA, 175 Da), has been demonstrated in animal models of pathology,^45^ and has more recently been demonstrated in human pathology.^46^ Gabr et al. reported in an advanced ^31^P MR diffusion spectroscopy study of human skeletal muscle that ‘In a time equal to the half-life of PCr in the CK reaction, PCr would diffuse an average distance of approximately 66 micrometres’.^47^ Although we could not find literature on such measurements in brain, we consider PCr as a diffusible, spatial buffer of energy reserve.

Unlike TBI, mitochondrial diseases showed *below*-normal PCr/ATP ratios in brains^48^ and heart muscle.^28,49^ Also, physiological adaptation (rather than disease) can give ‘below-normal’ PCr/ATP ratios, e.g. Sherpa heart muscle.^50^ In epilepsy, ipsilateral depression of PCr/ATP ratio occurred in the epileptogenic hippocampus, relative to contralateral ‘healthier’ hippocampus.^51^ Like TBI, *elevated* brain PCr/ATP ratios (above healthy controls) were reported in Parkinson’s disease,^52^ and in normal-appearing white matter in multiple sclerosis, attributed to diminished creatine kinase B activity.^53^ This supports the idea that inflammation in TBI brain might at least partly contribute to the elevated PCr/ATP ratio observed.

### Brain alkalosis following TBI

In the acute phase of TBI, patients’ brains overall were more alkaline compared to healthy controls. Sub-group analysis revealed this to be limited to patients who had unfavourable outcomes six months later (Figure 4). ^31^P MRS measures predominantly intracellular pH,^54^ estimated to represent 80% of total brain volume.^55,56^ Of this, we are likely detecting a composite of cytosolic and mitochondrial pH, as the Pi peaks from each pool do not appear separately resolved in our in vivo MRS. *Extracellular* acidification, measured with intracranial probes, is associated with metabolic derangement and increased TBI patient mortality.^24,25^ *Intracellular* acidification is seen in rodent ^31^P MRS studies of hyperacute major TBI,^57^ followed by a period of intracellular alkalosis.^26^ A ^31^P MRS study of recovering patients in the subacute/chronic phase of TBI found (intracellular) alkalosis of patients’ white matter.^27^ Our finding of brain intracellular alkalosis in acute-phase TBI patients with unfavourable outcome six months later suggests that acute-phase alkalosis occurs when physiology is severely deranged, rather than simply being a feature of recovery after injury. Brain alkalosis was found experimentally in rats 24–48 h after transient (8 min) forebrain ischaemia followed by reperfusion.^58^ In a clinical ^31^P MRS study of large-territory ischaemic stroke, hyper-acute brain acidification was reported (within 18 h of infarct), followed by brain alkalosis (by day 3) which persisted for 29 days^13^; these findings are supported by a later study of stroke patients 3–12 days after ictus.^42^ Infarction is an extreme tissue injury, so concurs with our finding of brain alkalosis in TBI patients who proceed to unfavourable outcomes – although our study demonstrates alkalosis in normal-appearing tissue on conventional and advanced MR imaging.

The interrelationships among intracellular pH, extracellular pH, and their biological implications are complex and incompletely understood. Mammalian cells strive to maintain an optimal intracellular pH and actively transport H^+^ ions extracellularly by several regulatory mechanisms, as they can only survive when intracellular pH is neutral or slightly alkaline.^59,60^ When astrocytes become alkalotic, their rate of glycolysis increases, likely through increased activity of the rate-limiting enzyme phosphofructokinase with an optimum around pH 7.2–7.3, and a steep pH dependence, falling dramatically at lower pH.^61,62^ Astrocyte glycolysis produces lactate; each lactate anion is co-transported by monocarboxylate transporters out of the cell accompanied by an H^+^ ion^59^ to ‘feed’ neurons in the model of the astrocyte-neuron lactate shuttle hypothesis.^63,64^ Upregulation of brain glycolytic activity was shown in acute severe TBI.^2,4,65,66^ If an increase in astrocytic lactate/H^+^ export was not matched by increased lactate/H^+^ uptake by neurons, extracellular acidosis would occur in the presence of intracellular alkalosis. Given the greater vulnerability of neurons compared to glia, this may explain the intracellular alkalosis we see, and the extracellular acidosis in other reports.^25,67^

Glial alkalosis may be self-regulated, or driven by neurons. Astrocytes can adapt their intracellular pH ‘set-point’ to be more alkaline,^68^ which increases their rate of glycolysis and synthesis of protein, DNA and RNA. This occurs in astrocytic tumours for cell division,^59,69^ and conceivably also for cellular repair in injured astrocytes. Several mechanisms regulate pH in astrocytes.^59^ Intracellular alkalosis may arise from the cell exporting H^+^ ions – including those generated by glycolysis. This may be via monocarboxylate transporters (see above) that do not consume ATP. However, another H^+^-extrusion pathway is via H^+^-ATPase pumps that directly consume ATP. If this were necessary for cells to maintain glycolysis, it may significantly reduce the net efficiency of glycolytic ATP production if mitochondrial function were impaired in the traumatised brain,^2,70^ as only two moles of ATP per mole of glucose are produced by ‘isolated glycolysis’. If NADH shuttling (e.g. malate-aspartate shuttle) from the cytosol into mitochondria is operational then further ATP molecules can ensue from glycolysis. The yield per mole of glucose metabolised fully to CO_2_ (by the combination of glycolysis, NADH shuttling and mitochondrial respiration) is theoretically 36–38 moles of ATP. However, the actual yield is considered somewhat lower.^71,72^ Upregulation of astrocyte glycolysis by intracellular alkalosis may also be activated by local neurons, as glial cytosolic alkalosis occurs in response to local neuronal activity.^73,74^ In TBI this may be a purposeful mechanism for neurons to induce additional metabolic support; or be a pathophysiological consequence of neuronal glutamate excitotoxic injury, associated with leakage of cytoplasmic elements from injured neurons.^75,76^

Alkali brain pH appears distinctive for patients who emerged with the worst clinical outcomes, concurring with the association between brain hyperglycolytic state and TBI mortality.^65^ This implies that after TBI, alkalosis is either caused by energy perturbation proceeding in the worst cases to an adaptive state of alkaline pH associated with abnormal cell biology and biochemistry, or that alkalosis is a distinct product of more catastrophic neuronal injury. The significant change in pH in patients with unfavourable outcome, with no change in patients with favourable outcome suggests that sedation did not affect brain pH, as all patients were treated effectively similarly.

A change in cell population, a proposed explanation for the elevation in PCr/ATP (see above), may also contribute to brain alkalisation. White matter alkalosis was attributed to reactive gliosis because regions of pathology containing more glial cells (e.g. low-grade astrocytomas) typically have a higher pH.^27^ Reactive gliosis may thus contribute to elevation of both pH and PCr/ATP in our study.

### Possible effects of sedation

Anaesthetising patients with propofol and midazolam sedation might have influenced our findings in TBI patients (Hypothesis 4, Figure 5(b)). Powerful sedatives such as pentobarbitone (pentobarbital)^77^ and high doses of diethyl ether, phenobarbital and sodium thiopentone^78^ increased cerebral PCr in rodents (assayed biochemically after extraction, not MRS). Conversely, light sedatives and analgesics had no effect on rat brain PCr concentration, and no sedative/anaesthetic agents (at any concentration) influenced cerebral ATP concentration in those studies.^39,77,78^ Pentobarbitone also decreased the TCA cycle rate in rats.^79^ Brain pH was not reported in those anaesthetic studies. The effects of propofol and midazolam on high-energy phosphates are unknown, but might resemble those of pentobarbitone and sodium thiopentone more than those of light sedation and analgesia. The elevation of PCr/total-mobile-phosphate was equivalent across our TBI outcome groups, so might be due to sedation. However, sedation/anaesthesia would not explain changes in ATP/total-mobile-phosphate (and pH, discussed below), as this decreased more in patients with unfavourable outcome, and all patients were treated essentially the same. Moreover, no changes in ATP were reported in the rodent sedation studies,^39,77,78^ and changes in PCr/ATP and pH were reported in subacute TBI patients, including those mechanically ventilated (without barbiturates, but presumably sedated) and those self-ventilating.^27^ Thus, although sedation may have influenced the changes in high-energy phosphates that we observe, it is probably less important than the effect of brain injury in accounting for differences in high-energy phosphates and pH in our study. Interestingly, brain PCr/ATP and pH both increased in a ^31^P MRS study of experimental (sheep) hypothermia.^80^ Although we did not use hypothermia, parallels may exist with TBI if ATP synthesis is faster than its utilisation and unused ATP increasingly transfers its high-energy phosphate energy potential to the PCr store.

### Ventilation and alkalosis

Increasing ventilation to lower PaCO_2_ can cause brain alkalosis, as shown by ^31^P MRS in self-hyperventilating healthy volunteers.^81^ In mechanically hyperventilated TBI patients, intracranial probes detected *extracellular* alkalosis.^82^ However, our TBI management protocol maintains arterial PaCO_2_ > 4 kPa, and the mean PaCO_2_ of our TBI patients was 4.8 kPa (range 4.3–7.4 kPa), equivalent to that expected in healthy subjects breathing normally. Interestingly, brain pH correlated inversely with arterial pH, apparently driven by arterial PaCO_2_ (Figure 3). Brain alkalosis was absent in our ventilated patients with favourable outcome who received similar treatment during MRS. Brain alkalosis was reported in TBI patients’ white matter in both self-ventilating and mechanically ventilated TBI patients sub-acutely post-injury.^27^

### Metabolic derangement in radiologically normal-appearing brain

When we excluded voxels with ≥5% radiological injury, the pattern of elevated PCr/ATP and elevated pH persisted in TBI brain (Figure 2), suggesting diffuse metabolic derangement throughout the brain. The difference in brain pH between all TBI patients and healthy controls was narrowly lost statistically, attributable to lower ‘n’ and the absence of change in patients with a favourable outcome ‘diluting’ the effect seen in patients with unfavourable outcome. Reported using ^1^H MRS six months post-TBI, the marker of neuronal integrity and density NAA/Cr was lower, and the cell turnover marker Cho/Cr higher in radiologically-normal-appearing brain, with the magnitude of change predicting patient outcome.^83^ Similar ^1^H MRS findings in another study appeared unrelated to abnormalities on conventional MR sequences.^66^ In the chronic phase of TBI, abnormalities were reported using ^1^H MRS in radiologically-normal-appearing patients’ thalami,^84^ frontal cortex^85^ and occipito-parietal cortex.^86^ Our ^31^P MRS findings of early metabolic abnormality in radiologically-normal-appearing brain after major TBI thus concur with previous ^1^H MRS reports, supporting the concept that microscopic whole-brain injury following TBI extends beyond macroscopic MRI-visible abnormalities.

### ^31^P MRS and clinical outcome

The relationship between the changes we observe in brain ^31^P MRS biochemistry and patient outcome suggests these changes represent underlying pathophysiology of brain injury, and that ^31^P MRS may be an early predictor of six-month outcome after major TBI. Patient outcome may correlate with some aspects of MRI-visible structural injury,^87^ but MRI-visible injury is unreliable alone as a predictor^88^ because of microscopic tissue damage without radiologically identifiable injury.^83,88,89^ ^1^H MRS characteristics of ‘microscopic’ tissue injury were identified acutely and sub-acutely after injury that correlate with clinical outcome^66^: recovery of NAA,^90^ NAA/Cr in the corpus callosum,^91^ and NAA/Cr in the brainstem.^92^ Our study is the first to demonstrate that acute-phase changes measured by ^31^P MRS relate to clinical outcomes six months later. Brain alkalosis only occurred in patients with ultimately unfavourable outcomes. Importantly, this remained true when injured voxels were excluded, suggesting ^31^P MRS utility in addition to ^1^H MRS structural imaging. Of the three patients with unfavourable outcome and severe brain alkalosis (P03, P04, P05), their predicted risks of unfavourable outcome at six months were respectively 31%, 74% and 86%, on the CRASH head injury prognosis calculator (Supplementary Table 2),^93^ suggesting that patient P03 suffered metabolic injury unapparent to current predictive models. Early warning from ^31^P MRS measurement of alkalosis that a patient may be heading towards unfavourable outcome will maximise opportunity for intervention to improve outcome. Furthermore, patient P02 *without* pronounced brain alkalosis emerged with favourable (GOS-E 5) outcome, despite CRASH prediction of 90% risk of unfavourable outcome. Although PCr/ATP did not reliably discriminate between TBI patient outcome groups, ATP/total-mobile-phosphate was lower in patients with unfavourable outcome than patients with favourable outcome. Again, this persisted when radiologically injured voxel data were excluded (Figure 4). ^31^P MRS may thus help early recognition of patients who will emerge better than conventionally predicted. Our promising findings merit further, larger studies to confirm these relationships between acute ^31^P MRS and 6-month outcome, and determine whether the technique might augment existing (non-MRS) outcome prognostication.^93^

### Strengths and limitations

^31^P comprises 100% of all naturally occurring phosphorus atoms, so artificial enrichment is unnecessary for MR, which is non-invasive. Due to technical constraints of in vivo MRS, absolute quantification of concentrations is very difficult to achieve accurately, so we used PCr/ATP ratio; enabling reliable comparisons for statistical analysis. For good sensitivity within a practicable scan duration, we used 2.5 × 2.5 × 2.5 cm^3^ voxels. The central eight voxels were chosen as they represented a large volume of subjects’ brains, whilst avoiding signal contamination from bone, muscle and scalp. Only 17 central eight voxels contained >5% injury, too few for meaningful subgroup statistical analysis.

In vivo MRI/MRS of sedated, ventilated patients in the acute phase of secondary brain injury following major TBI is challenging, and ^31^P MRS, although non-invasive, safe, and not excessive on scan time, is not yet routinely used. Our promising results in this small cohort (13 patients, 10 controls) merit larger studies, including uninjured anaesthetised brain, to elucidate fundamental biochemical abnormalities following major TBI and establish the prognostic power of acute-phase ^31^P MRS.

## Conclusions

Here we have shown that clinical in vivo ^31^P MRS reveals significant changes in brain high-energy phosphate metabolism acutely after major TBI, indicating that a change in brain energy state accompanies known changes in brain metabolism.^2^ The higher energy-store status (PCr/ATP) found in TBI patients compared to healthy controls is perhaps surprising, given the conventional view of ‘energy crisis’ in TBI, but appears to be due to both a relative increase in PCr and a relative fall in ATP, within the combined pool of total-mobile-phosphate. Among various hypotheses, upregulation of resident microglia and migratory influx of inflammatory cells with higher PCr/ATP ratio are likely, with possible variation in energy status dependent on the cell type and susceptibility to injury. Acute TBI and inflammation are usually regarded as associated with (extracellular) ‘acidosis’, but we show for the first time that brain (intracellular) alkalosis occurs in the acute phase of TBI. This appears limited to patients who emerge with unfavourable outcome after six months, suggesting acute-phase raised intracellular pH is important pathophysiologically in TBI. These changes in PCr/ATP and pH are present in radiologically normal, ‘uninjured’ tissue on MR sequences sensitive for injury, suggesting widespread metabolic derangement occurs throughout the brain after major TBI, not just in lesions. Although we primarily used patient outcome to help understand these biochemical changes, brain pH shows potential as an early predictor of patient outcome after major TBI. No patient with a mean brain pH ≥ 7.05 had a favourable outcome. Combining voxel-based ^31^P MRS brain pH measurement with existing predictive TBI outcome models may improve accuracy. Further study of ^31^P MRS in TBI is merited to explore its full potential in evaluating brain injury, response to therapy, and how these correlate with outcome.

## Supplemental Material

Supplemental material for Phosphorus spectroscopy in acute TBI demonstrates metabolic changes that relate to outcome in the presence of normal structural MRI Click here for additional data file. Supplemental material for Phosphorus spectroscopy in acute TBI demonstrates metabolic changes that relate to outcome in the presence of normal structural MRI by Matthew G Stovell, Marius O Mada, T Adrian Carpenter, Jiun-Lin Yan, Mathew R Guilfoyle, Ibrahim Jalloh, Karen E Welsh, Adel Helmy, Duncan J Howe, Peter Grice, Andrew Mason, Susan Giorgi-Coll, Clare N Gallagher, Michael P Murphy, David K Menon, Peter J Hutchinson and Keri LH Carpenter in Journal of Cerebral Blood Flow & Metabolism
