# Supplementary material for: Phosphorus spectroscopy in acute TBI demonstrates metabolic changes that relate to outcome in the presence of normal structural MRI
Source: J Cereb Blood Flow Metab. 2018 Sep 18;40(1):67–84. doi: 10.1177/0271678X18799176 (PMC6927074; doi:10.1177/0271678X18799176)
Supplement: Supplemental material for Phosphorus spectroscopy in acute TBI demonstrates metabolic changes that relate to outcome in the presence of normal structural MRI [file Supplemental_material10.pdf]

Supplementary Table 1. Demography of healthy controls

Subject number	Age group	Sex
H01	50-65	M
H02	20-34	F
H03	50-65	M
H04	35-49	F
H05	20-34	F
H06	20-34	M
H07	20-34	M
H08	35-49	F
H09	35-49	M
H10	50-65	F

M: male, F: female, Age group in years. Healthy controls were selected to match the age group of the TBI patients.

Supplementary Table 2. CRASH predictions for the TBI patients

**Subject number**	**CRASH prediction of unfavourable outcome**
P01	42 %
P02	90 %
P03	31 %
P04	74 %
P05	86 %
P06	22 %
P07	39 %
P08	31 %
P09	69 %
P10	83 %
P11	35 %
P12	28 %
P13	46 %

Risk of unfavourable outcome at six months calculated using online head injury prognosis calculator based on data from the CRASH trial (Corticosteroid Randomisation After Significant Head Injury). http://www.trialscoordinatingcentre.lshtm.ac.uk/Risk%20calculator/index.html
